# A Systematic Review of Pathogenic *COL4A5* Variants and Proteinuria in Women and Girls With X-linked Alport Syndrome

**DOI:** 10.1016/j.ekir.2022.08.021

**Published:** 2022-08-29

**Authors:** Joel T. Gibson, Mikayla de Gooyer, Mary Huang, Judy Savige

**Affiliations:** 1 Department of Medicine (Melbourne Health and Northern Health), University of Melbourne, Parkville, Victoria, Australia; 2 The Royal Melbourne Hospital, Parkville, Victoria, Australia

**Keywords:** Alport syndrome, *COL4A5*, female, kidney failure, proteinuria

## Abstract

**Introduction:**

Women and girls with X-linked Alport syndrome have a risk of disease progression that is difficult to predict. This systematic review examined whether proteinuria correlated with genotype and disease severity in this population.

**Methods:**

PubMed and Scopus were searched for manuscripts from the past 20 years with “*COL4A5*,” “female,” “proteinuria” and related terms. Genotypes and clinical data for women and girls with pathogenic heterozygous *COL4A5* variants were extracted. Features were then compared between females with proteinuria or without proteinuria; and genotype-phenotype correlations for age at proteinuria and kidney failure determined.

**Results:**

Three-hundred sixty-six women and girls with *COL4A5* variants and a median age of 29 years (interquartile range 15–46) were identified. Eighty-eight (24%) had large rearrangements or truncating variants, 63 (17%) had splicing variants, and 215 (59%) had missense changes. In all, 236 (64%) had proteinuria, 56 (16%) had kidney failure, 40 (16%) had a hearing loss, and 15 (7%) had ocular abnormalities. Women and girls with proteinuria were more likely to have large rearrangements or truncating variants (*P* = 0.005), and less likely to have missense changes (*P* = 0.0002). Those with proteinuria were also more likely to develop kidney failure (*P* < 0.0001). Women and girls with truncating, large or splicing variants developed proteinuria earlier than those with missense changes (*P* = 0.001, *P* < 0.0001 respectively). Those whose proteinuria was detected before the age of 15 progressed to kidney failure sooner (*P* < 0.0001).

**Conclusion:**

Proteinuria correlates with a more severe genotype in women and girls with X-linked Alport syndrome and is an indicator of disease severity and likely progression.

## Graphical abstract

**Figure:**
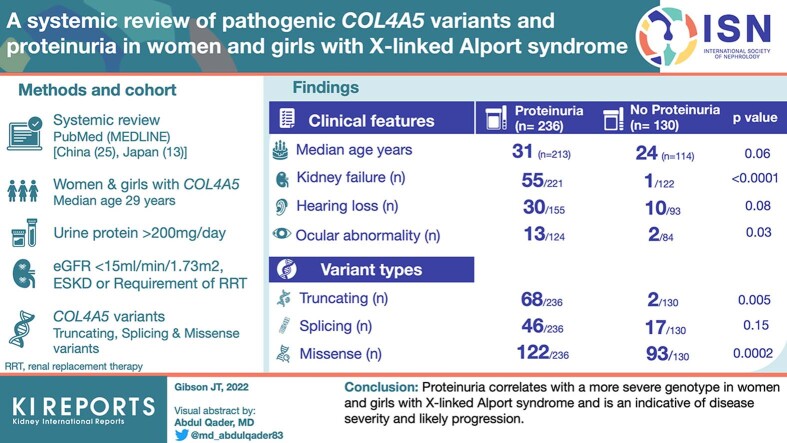

Alport syndrome is the commonest genetic kidney disease, and the second commonest inherited cause of kidney failure after autosomal dominant polycystic kidney disease.^1^^,^^2^ Alport syndrome results from pathogenic variants in the *COL4A3*-*COL4A5* genes, which encode the collagen IV α3-α5 chains.^3^^,^^4^ These chains trimerize to form the collagenous networks in the basement membranes of the kidney, ear and eye.^5^

X-linked Alport syndrome due to pathogenic *COL4A5* variants is the commonest inheritance pattern that causes severe disease. Ninety percent of males develop end-stage kidney failure before the age of 40, and many also have sensorineural hearing loss and ocular abnormalities.^6^, ^7^, ^8^ The clinical features in women and girls with X-linked disease are more variable, with kidney failure and extrarenal abnormalities occurring less often and later, if at all.^9^^,^^10^

Men and boys with X-linked Alport syndrome demonstrate a strong genotype-phenotype correlation. Males with large deletions or truncating variants develop kidney failure earlier than those with missense changes.^6^, ^7^, ^8^ Splicing variants have an effect intermediate between truncating and missense changes. Gly substitutions may be further stratified. The intermediate collagenous domain of the collagen IV α5 chain is “interrupted” by 22 short noncollagenous sequences. Gly substitutions directly adjacent to the interruptions are associated with later onset kidney failure^11^ because the interruptions allow more space for differently-sized amino acids than the relatively inflexible collagen helix. Gly substitutions with mildly destabilizing residues such as Ala, Ser and Cys also result in a later age at kidney failure than substitutions with highly destabilizing residues such as Arg, Val, Glu, Asp and Trp.^11^ There are similar genotype-phenotype correlations for hearing loss and ocular abnormalities in males.^6^, ^7^, ^8^^,^^11^^,^^12^

Previous studies of women and girls with X-linked Alport syndrome have not identified a genotype-phenotype correlation for kidney failure, hearing loss or ocular abnormalities.^9^^,^^13^^,^^14^ In one series of 349 affected women and girls, the risk of kidney failure was not increased with large rearrangements, splice site or missense variants.^9^ However, those with proteinuria or hearing loss still had the highest risk of kidney failure.

Genotype-phenotype correlations for kidney failure in women and girls have been complicated by the smaller numbers of affected individuals and because of random X-chromosome inactivation, where the X-chromosomes are expressed in a mosaic pattern.^10^ In mouse models of X-linked Alport syndrome, preferential inactivation of the X-chromosome with the normal allele results in worse kidney function and lower rates of survival.^15^ Nevertheless, studies in humans are difficult to interpret, in part because X-inactivation patterns vary in different affected tissues and the peripheral blood cells that are usually examined.^16^, ^17^, ^18^, ^19^

The lack of genotype-phenotype correlation in women and girls with X-linked Alport syndrome manifests also in the large intrafamilial variability of the age at kidney failure, compared with male relatives.^9^ Any genotype-phenotype correlation is considered less pronounced in females than in males, and that therefore larger cohorts are required to demonstrate a relationship. Other causes of kidney failure, such as hypertension, pre-eclampsia, smoking and obesity may also contribute to kidney function impairment.^20^

Nevertheless, proteinuria has long been recognized as a risk factor for kidney failure in Alport syndrome,^9^ and a recent single-center study of 24 women and girls found that those with “severe” *COL4A5* variants (large deletions, rearrangements, truncating, splice site, and digenic variants) were more likely to have proteinuria and impaired kidney function than those with missense changes.^14^ If confirmed, this has clinical implications because the genotype can be used to identify the risk of disease progression. Currently, treatment in women and girls with X-linked Alport syndrome is only commenced after the detection of proteinuria.^21^ Confirmation of a genotype-phenotype correlation represents an argument for more careful monitoring of the female with a severe variant and the consequent earlier detection of proteinuria and institution of treatment.

This systematic review has examined genetic and clinical data from all women and girls with X-linked Alport syndrome reported in the previous 20 years in order to determine a genotype-phenotype correlation.

## Methods

### Systematic Search

PubMed (MEDLINE) and Scopus (both accessed 30 March 2022) were searched for abstracts, citations and keywords from manuscripts published since 2002 that included “*COL4A5*,” “female”, “proteinuria,” and related terms (Supplementary Table S1, Supplementary Table S2). Manuscripts published before 2002, in languages other than English, or where the full texts were not available were excluded (Supplementary Figure S1). Each manuscript, including any supplementary material, was then screened independently by 2 reviewers, and relevant data (genotype, age, proteinuria level, kidney function, hearing loss, ocular abnormalities) for all women and girls with a heterozygous pathogenic *COL4A5* variant noted. Manuscripts where data were not described at the individual level, or that reported previously-published cases were excluded. No automated tools were used.

### Inclusion Criteria

Only women and girls with *COL4A5* variants assessed as “Pathogenic” or “Likely Pathogenic” by the authors, and where the results of testing for proteinuria were known were included. Other female family members who had not undergone genetic testing themselves were included only if they were obligate carriers. Those with multiple pathogenic *COL4A3*–*COL4A5* variants, homozygous variants, or demonstrated to have somatic mosaicism, were excluded.

Proteinuria was defined as a urinary protein level greater than 200 mg/day (or 200 mg/l), a urinary protein creatinine ratio greater than 0.2 g/g, a dipstick protein reading of 1+ or more, or a clinical diagnosis of proteinuria. Kidney failure was defined as an estimated glomerular filtration rate less than15 ml/min per 1.73 m^2^, CKD stage 5, a clinical diagnosis of end-stage kidney failure or a requirement for dialysis or kidney transplantation. Hearing loss was identified by manuscript authors most often by clinical questioning or on an audiogram result.

### Variants

Variants were categorized into 3 groups as follows for analysis:

• Large variants (deletions, insertions and rearrangements) affecting more than 20 nucleotides and truncating variants (nonsense and frameshift).
• Canonical splice site variants (+/− 1 or 2) and other exonic or intronic variants that had been demonstrated experimentally to affect splicing.
• Missense variants (Gly or otherwise), but excluding apparent missense variants subsequently demonstrated to affect splicing which were grouped with the splicing variants. Gly missense variants were also examined separately to determine the effect of location immediately adjacent to a noncollagenous interruption or terminus,^22^ and the degree of instability caused by the residue replacing Gly (mildly destabilizing: Ala, Ser, Cys; or highly destabilizing: Arg, Val, Glu, Asp, Trp).^23^

### Analysis

Women and girls with proteinuria were compared with those without proteinuria to determine any differences in pathogenic variant type, and age, kidney function, or extrarenal features.

Survival analysis was used to determine genotype-phenotype correlation for the age at onset of proteinuria. The reported age at proteinuria likely represented the age when proteinuria was first detected rather than the age at onset which may have been much earlier. These terms are used here interchangeably for the purpose of analysis.

Survival analysis was also used to determine whether proteinuria was detected at a younger age in countries with mass urinary screening programs for school-aged children, such as Japan and South Korea, compared with other countries.

Finally, genotype-phenotype correlations for age at kidney failure were examined, and the age at kidney failure compared between women and girls where proteinuria was first detected before the age of 15 and where it occurred later.

For all survival analyses, women and girls who did not have proteinuria or kidney failure at their most recent examination, were included as censored data points. Those where the age at first report of proteinuria or kidney failure was not recorded were excluded.

### Statistical Analysis

All statistical analyses were performed using R (version 3.6.2, https://www.R-project.org/), and included the survival and survminer packages.^24^, ^25^, ^26^ Categorical data were compared using Fisher’s exact test, and continuous data using the Mann-Whitney U test. Survival curves were produced using the Kaplan-Meier method, and compared using the log-rank test. Covariates in the survival analysis were assessed using a Cox proportional hazards model. A *P*-value less than 0.05 was considered significant, and a *P*-value less than 0.1 but greater than 0.05 was considered a trend.

## Results

### Cohort Characteristics

Two-hundred fifty-one unique manuscripts were identified from the systematic search, of which 89 were included in the final study (Supplementary Figure S1, Supplementary Table S3). These were mostly from East Asian (*n* = 44) or European (*n* = 35) populations, and the most frequently represented countries were China (*n* = 25) and Japan (*n* = 13) (Supplementary Table S4).

Variants and data were available for 366 women and girls with X-linked Alport syndrome, who had a median age of 29 years (interquartile range 15–46 years, *n* = 327).

### Variant Types

Missense variants were the most common type (*n* = 215, 59%), followed by truncating variants or large rearrangements (*n* = 88, 24%) and splicing variants (*n* = 63, 17%) (Table 1). Three individuals with in-frame deletions with fewer than 20 nucleotides were excluded from further analysis.

**Table 1:** Clinical features and variant types in 366 females with X-linked Alport syndrome

Clinical features and variant type	Proteinuria (*n* = 236)	No proteinuria (*n* = 130)	*P*-value
Median age (IQR) (yr)	31 (16–47) (*n* = 213)	24 (14–42) (*n* = 114)	0.06
Kidney failure (n, %)	55/221 (25%)	1/122 (1%)	<0.0001^a^
Hearing loss (n, %)	30/155 (19%)	10/93 (11%)	0.08
Ocular changes (n, %)	13/124 (10%)	2/84 (2%)	0.03^a^
Variant types			
Truncating and large variants (n, %)	68/236 (29%)	20/130 (15%)	0.005^a^
Splicing variants (n, %)	46/236 (19%)	17/130 (13%)	0.15
Missense variants (n, %)	122/236 (52%)	93/130 (72%)	0.0002^a^

IQR, interquartile range.

a Significant values.

### Clinical Features

A total of 236 women and girls had proteinuria (64%) (Table 1). On average, those with proteinuria were 7 years older than those without proteinuria (*P* = 0.06). Fifty-six (*n* = 56/343, 16%) had kidney failure, which was almost exclusively in those who were previously noted to have proteinuria (*n* = 55, 98%; *P* < 0.0001).

Extrarenal features were reported uncommonly. In total, 40 women and girls had a hearing loss (*n* = 40/248, 16%), and there was a trend to proteinuria being associated with the hearing loss (*P* = 0.08). Fifteen women and girls had ocular abnormalities (*n* = 15/208, 7%), including lenticonus, a giant macular hole, and other unspecified changes. Ocular changes were more common in those with proteinuria (*P* = 0.03).

Women and girls with proteinuria were more likely to have truncating or large variants than those without proteinuria (*P* = 0.005), and were less likely to have missense variants (*P* = 0.0002). The proportion of splicing variants did not differ between those with proteinuria or those without (*P* = 0.15).

Considering how different the median ages were between the groups with proteinuria and those without proteinuria, the analysis was repeated using an age-matched subgroup of the proteinuric cohort. Subgroups of 160 females with proteinuria were generated randomly until a subgroup with a median age within 1 year of that of the group without proteinuria was found. The analysis was repeated and similar results were obtained for both the clinical features and variant types (Supplementary Table S5).

### Age at Proteinuria

**Figure 1:**
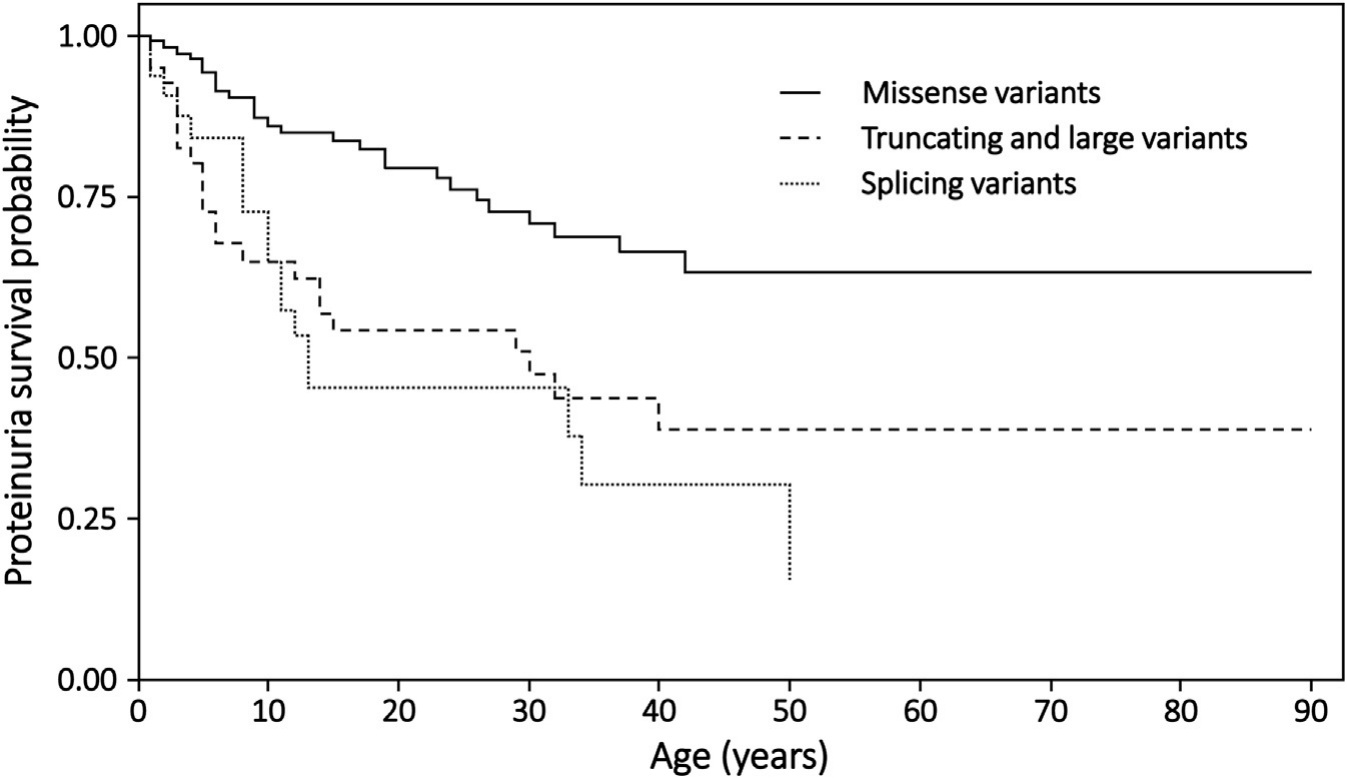
Proportion of females without proteinuria, stratified by variant type (p=0.0001). Missense variants (n_total_ = 108, n_prot_ = 27); truncating/large variants (n_total_ = 41, n_prot_ = 22, median=30 years); splicing variants (n_total_ = 32, n_prot_=18, median = 13 years). The “missense variant” survival curve does not pass below 0.5, so that the median age at survival is not available. Pairwise comparisons: missense versus splicing (*P* < 0.0001); missense versus truncating/large (*P* = 0.001); splicing versus truncating/large (*P* = 0.51). Censored data points are not shown.

In total, 181 females, including 67 with proteinuria, were included in the proteinuria survival analysis. Because many survival curves did not fall below 0.5, the median ages at detection of proteinuria for each subgroup could not always be calculated. Overall, the median age at detection of proteinuria was 50 years (range 1–50), but this differed by variant type (*P* = 0.0001) (Figure 1). Women and girls with truncating or large variants (*n*_*total*_ = 41, *n*_*prot*_ = 22, median = 30 years, *P* = 0.001) or with splicing variants (*n*_*total*_ = 32, *n*_*prot*_ = 18, median = 13 years, *P* < 0.0001) had an earlier age at detection of proteinuria than those with missense variants (*n*_*total*_ = 108, *n*_*prot*_ = 27). There was no difference in age at detection of proteinuria between those with truncating or large variants and those with splicing variants (*P* = 0.51). For all variant types, the first detection of proteinuria after the age 40 years was rare. Examining missense variants in isolation, there was no difference observed for women and girls with Gly substitutions (*n*_*total*_ = 83, *n*_*prot*_ = 23) compared with non-Gly substitutions (*n*_*total*_ = 25, *n*_*prot*_ = 4, *P* = 0.12) (Supplementary Figure S2), but the age at proteinuria was known for only 4 individuals with non-Gly substitutions.

Considering only Gly substitutions, there was no difference in the age at proteinuria detection for Gly substitutions adjacent to noncollagenous regions (*n*_*total*_ = 19, *n*_*prot*_ = 4, median = 42 years) compared with other Gly substitutions (*n*_*total*_ = 60, *n*_*prot*_ = 15, *P* = 0.45) (Supplementary Figure S3). Interestingly, the youngest age at detection of proteinuria for a woman or girl with a Gly substitution adjacent to a noncollagenous region was 23 years, which was much later than for most females with Gly substitutions not adjacent to a noncollagenous region, but the sample sizes were probably too small to demonstrate any difference.

Considering the identity of the substituting residue, there was also no difference between substitutions with mildly (Ala, Ser, Cys) (*n*_*total*_ = 13, *n*_*prot*_ = 3) or highly destabilizing residues (Arg, Val, Glu, Asp, Trp) (*n*_*total*_ = 66, *n*_*prot*_ = 16, *P* = 0.83) (Supplementary Figure S4), but again the sample sizes were small.

The age at proteinuria detection was much younger in women and girls from countries with mass urinary screening programs, such as Japan and South Korea (*n*_*total*_ = 20, *n*_*prot*_ = 14, median = 10 years), than in other countries (*n*_*total*_ = 161, *n*_*prot*_ = 53,*P* < 0.0001) (Supplementary Figure S5). The median age at detection of proteinuria in the Japanese and South Korean cohorts was 10 years, whereas the proportion of those without proteinuria from other countries was still above 0.5 at 50 years. Nevertheless, inclusion of this covariate in a Cox proportional hazards model demonstrated that truncating or large variants, and splicing variants were still both associated with earlier ages at proteinuria detection than missense variants (*P* = 0.01, *P* = 0.002, respectively), so that this was unlikely to have biased these results.

### Age at Kidney Failure

**Figure 2:**
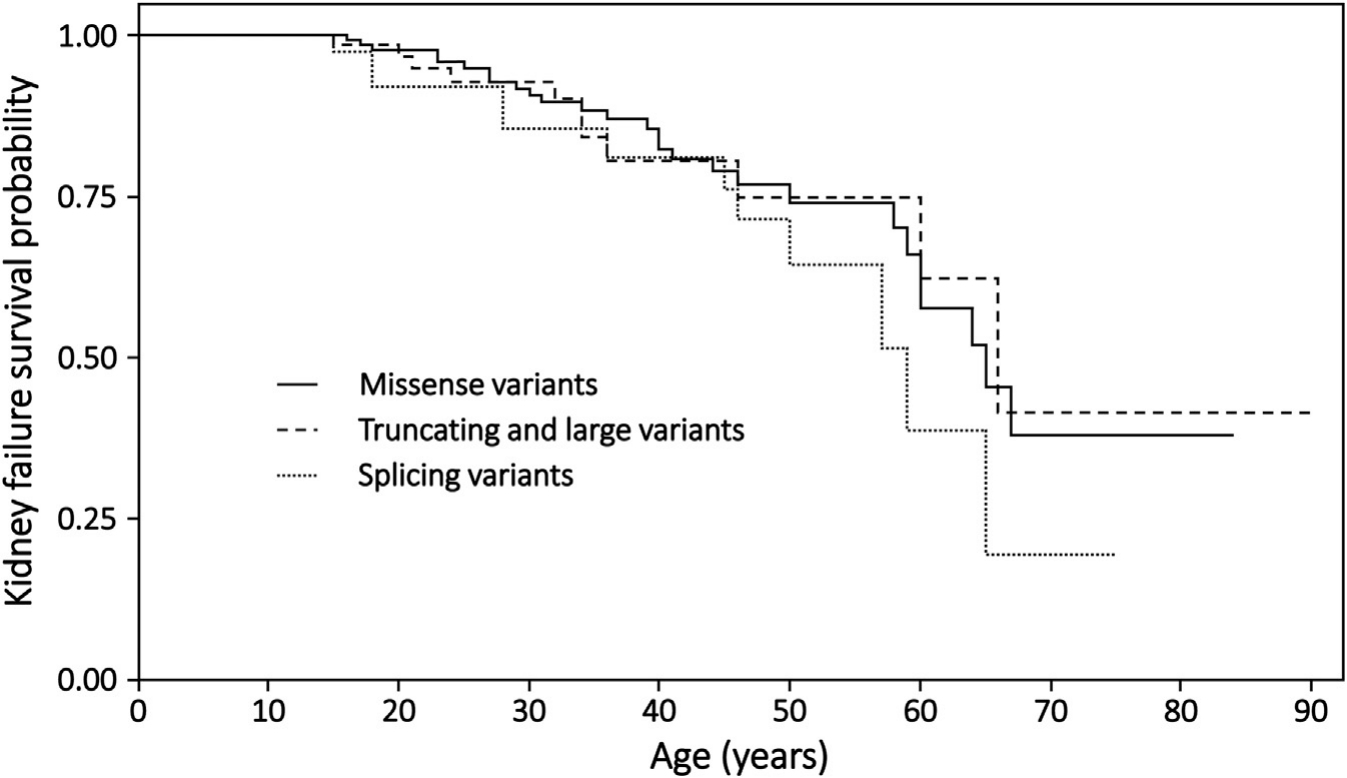
Proportion of females without kidney failure, stratified by variant type (*P* = 0.41). Missense variants (n_total_ = 172, n_KF_ = 27, median = 65 years); truncating/large variants (n_total_ = 77, n_KF_ = 11, median = 66 years); splicing variants (n_total_ = 56, n_KF_ = 12, median = 59 years). Censored data points are not shown.

In total, 305 women and girls, including 50 with kidney failure, were included in the survival analysis. Overall, the median age at kidney failure was 65 years (range 15–67) years. As demonstrated in previous studies of X-linked Alport syndrome in women and girls, there was no genotype-phenotype correlation for age at kidney failure (*P* = 0.41) (Figure 2).

**Figure 3:**
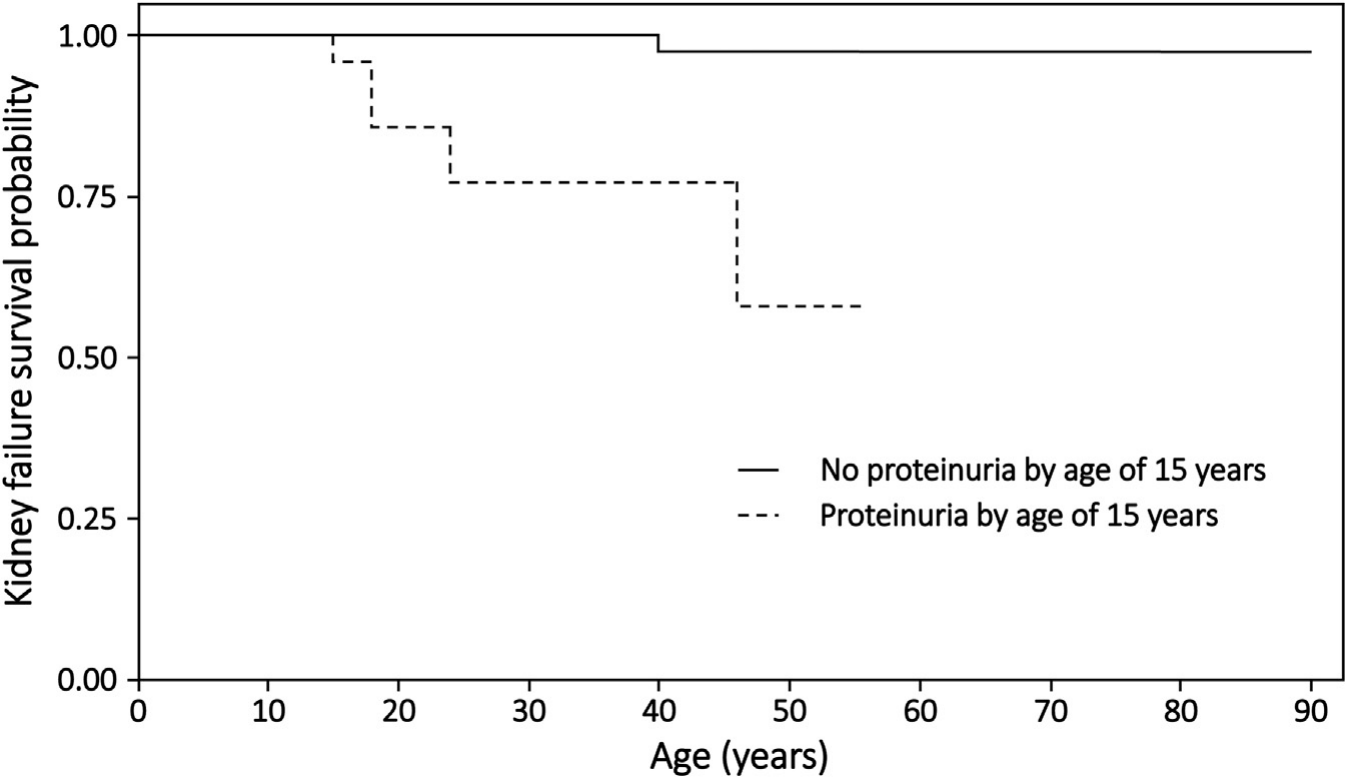
Proportion of females without kidney failure, stratified by age at onset of proteinuria (*P* < 0.0001). No proteinuria by 15 years old (n_total_ = 92, n_KF_ = 1); proteinuria by 15 years old (n_total_ = 65, n_KF_ = 5). Neither survival curve passes below 0.5, so that the median ages at survival are not available. Censored data points are not shown.

Only 6 females with kidney failure had both age at proteinuria and age at kidney failure recorded. Despite this small sample size, those with proteinuria onset before age 15 years (n_total_ = 65, n_KF_ = 5) were observed to progress to kidney failure sooner than those without proteinuria at the age of 15 (n_total_ = 92, n_KF_ = 1, *P* < 0.0001) (Figure 3).

## Discussion

Women and girls with X-linked Alport syndrome have a variable and typically milder phenotype than affected males. Previous genotype-phenotype studies in females have not found a clear correlation with disease progression, except for a small single-center study that included digenic variants.^14^^,^^27^ The present systematic review has demonstrated a genotype-phenotype correlation in affected women and girls with X-linked Alport syndrome, and that proteinuria represents a marker for development of kidney failure and extrarenal complications.

Women and girls with proteinuria were more likely to have truncating or large variants than those without proteinuria, and less likely to have missense variants. Truncating or large variants, together with splicing variants, were also associated with an earlier age at detection of proteinuria than missense changes. This association was confirmed in an age-matched subset from within the cohort. Furthermore, almost all women and girls with X-linked Alport syndrome and kidney failure had preceding proteinuria. The exception was a 90-year-old woman with the hypomorphic p.Gly624Asp variant.^28^ In addition, girls with proteinuria detected before age 15 years progressed to kidney failure sooner than those without proteinuria, consistent with early-onset proteinuria reflecting more severe disease. Overall kidney failure and extrarenal features were more common in women and girls with proteinuria, suggesting that proteinuria identifies those at risk of severe disease.

This cohort, although derived from multiple reports, was typical genetically and clinically of X-linked Alport syndrome. The likelihood of variant types approximated to the expected frequencies of 50% for severe variants and 50% for milder changes.^29^ In total, 56 of the women (16%) developed kidney failure at a median age of 65 years, consistent with earlier studies that found that 15% to 30% of women had kidney failure by the age of 60.^9^^,^^30^ However, overall extrarenal features such as hearing loss and ocular abnormalities were less common than reported in European cohorts,^9^^,^^31^ but more frequent than in younger patients from Japan.^13^

A potential source of bias in the present study was the inclusion of the rarer large deletions that are associated with distinctive clinical features such as leiomyomata and intellectual disability.^32^, ^33^, ^34^, ^35^, ^36^ These individuals were likely to have been recognized sooner and monitored more closely, potentially leading to the detection of proteinuria at a younger age. However, the number of such cases was likely too small to have affected our overall conclusions. In addition, splicing variants which have no distinctive clinical features were also associated with an earlier age at proteinuria than missense variants.

Proteinuria was detected at a younger age in Japan and South Korea that have mass urinary screening programs for school age children. This means that the median age at proteinuria in these populations is a more accurate reflection of the age at proteinuria onset than in countries where women and girls may have undetected proteinuria for years.^13^ Where there are no screening programs, women and girls with X-linked Alport syndrome are often only tested after an affected male relative’s diagnosis, which also contributes to the later age at proteinuria detection. Treatment of women and girls with X-linked Alport syndrome is commonly only commenced after the demonstration of proteinuria, so that earlier detection through regular screening means that treatment to delay kidney failure may be commenced earlier.

It is likely that proteinuria is both a marker for, and a contributor to, disease progression in Alport syndrome. Proteinuria probably reflects the presence of secondary focal and segmental glomerulosclerosis,^37^ where the underlying pathology is podocyte loss from the defective basement membrane.^38^^,^^39^ The epithelial loss is not confined to the kidney, but also explains the extrarenal manifestations in the cornea and retina.^40^ In addition, a canine model of Alport syndrome suggests that a reduced nephron number at birth contributes to secondary focal and segmental glomerulosclerosis from hyperfiltration.^41^

Nevertheless, genotype still did not correlate with age at kidney failure in women and girls with X-linked Alport syndrome despite correlations between genotype and age at proteinuria, and between age at proteinuria and kidney failure onset. This contrasts with X-linked disease in males, where a direct genotype-phenotype relationship has been demonstrated repeatedly in much smaller cohorts. The requirement for a larger cohort in females suggests that the genotype is less important in determining kidney failure than in males, possibly due to X-chromosome inactivation, and that more podocytes must be damaged to result in kidney failure than to cause proteinuria. Other contributions such as poor blood pressure control and genetic modifiers may also play a role.

The current recommendation for managing women and girls with X-linked Alport syndrome is that they are treated from the detection of proteinuria.^21^ The genotype-phenotype correlation described here suggests that women and girls with severe pathogenic variants should be monitored more closely for proteinuria so that treatment is commenced as early as possible.^42^ However, in women, the risks of renin-angiotensin-blockade must also be acknowledged, especially the risks in early pregnancy. The demonstration of a severe variant in a woman also represents a further argument against her acting as a kidney donor.

This study has demonstrated that proteinuria is a marker of disease severity and progression in women and girls with X-linked Alport syndrome, and furthermore that severe pathogenic variants are associated with earlier-onset proteinuria, disease progression, and extrarenal features. Nevertheless, it is still not possible to predict the age at kidney failure in women with X-linked Alport syndrome from the genetic variant as accurately as in men.^42^

## Disclosur**e**

All the authors declared no competing financial or non-financial interest.

## Supplementary Material

Supplementary File (PDF)

**Figure S1.** PRISMA flow chart for study selection

**Figure S2.** Proportion of females without proteinuria, stratified by missense variant type

**Figure S3.** Proportion of females without proteinuria, stratified by location of Gly substitution

**Figure S4.** Proportion of females without proteinuria, stratified by the degree of instability caused by the residue replacing Gly

**Figure S5.** Proportion of females without proteinuria, stratified by location of study

**Table S1.** PubMed search strategy

**Table S2.** Scopus search strategy

**Table S3.** List of articles included in this systematic review

**Table S4.** Location of studies included in this systematic review

**Table S5.** Clinical features and variant types in 274 age-matched females with X-linked Alport syndrome

**PRISMA Checklist**.
