# Supplementary material for: A Systematic Review of Pathogenic COL4A5 Variants and Proteinuria in Women and Girls With X-linked Alport Syndrome
Source: Kidney Int Rep. 2022 Aug 29;7(11):2454–61. doi: 10.1016/j.ekir.2022.08.021 (PMC9751687; doi:10.1016/j.ekir.2022.08.021)
Supplement: Supplementary File (PDF) [file mmc1.pdf]

Supplementary Material

Supplementary Table S1. PubMed search strategy

Supplementary Table S2. Scopus search strategy

Supplementary Table S3. List of manuscripts included in this systematic review

Supplementary Table S4. Geographic location of studies included in this systematic review

Supplementary Table S5. Clinical features and variant types in 274 age-matched females with X-linked Alport syndrome

Supplementary Figure S1. PRISMA flow chart for study selection

Supplementary Figure S2. Proportion of females without proteinuria, stratified by missense variant type

Supplementary Figure S3. Proportion of females without proteinuria, stratified by location of Gly substitution

Supplementary Figure S4. Proportion of females without proteinuria, stratified by the relative instability of the residue replacing Gly

Supplementary Figure S5. Proportion of females without proteinuria, stratified by geographic location of study

PRISMA checklist

Supplementary Table S1. PubMed search strategy.

**#1**	"COL4A5 protein, human"[Supplementary Concept] OR COL4A5[tw]
**#2**	"Proteinuria"[Mesh] OR "Nephrotic Syndrome"[Mesh] OR "Urinalysis"[Mesh] OR “Phenotype”[Mesh] OR Proteinuri*[tw] OR Nephrotic[tw] OR Albuminuria[tw] OR Microalbuminuria[tw] OR Urinalysis[tw] OR Dipstick[tw] OR Phenotyp*[tw]
**#3**	"Female"[Mesh] OR “Family”[Mesh] OR “Pedigree”[Mesh] OR Female*[tw] OR Woman[tw] OR Women[tw] OR Girl*[tw] OR Mother*[tw] OR Maternal[tw] OR Sister*[tw] OR Daughter*[tw] OR Aunt*[tw] OR Niece*[tw] OR Grandmother*[tw] OR Famil*[tw] OR Pedigree*[tw] OR Gender*[tw] OR Sex[tw] OR Sexes[tw]
**#4**	#1 AND #2 AND #3

Supplementary Table S2. Scopus search strategy.

**#1**	TITLE-ABS-KEY (col4a5)
**#2**	TITLE-ABS-KEY (proteinuri* OR nephrotic OR albuminuria OR microalbuminuria OR urinalysis OR dipstick OR phenotyp*)
**#3**	TITLE-ABS-KEY (female OR woman OR girl OR mother OR maternal OR sister OR daughter OR aunt OR niece OR grandmother OR famil* OR pedigree OR gender OR sex)
**#4**	#1 AND #2 AND #3

Supplementary Table S3. List of articles included in this systematic review.

Authors	Year	Title
Abe et al.	2016	A novel mutation in a Japanese family with X-linked Alport syndrome
Allred et al.	2015	Phenotypic heterogeneity in females with X-linked Alport syndrome
Almokali et al.	2021	The frequency of genetic mutations in pediatric patients diagnosed with nephrotic syndrome: A single-center retrospective study in Saudi Arabia
Ars et al.	2005	Male-to-male transmission of X-linked Alport syndrome in a boy with a 47,XXY karyotype
Baikara et al.	2015	A novel mutation in a Kazakh family with X-linked Alport syndrome
Bałdyga et al.	2021	Complex phenotypic presentation of syndromic hearing loss deciphered as three separate clinical entities: How genetic testing guides final diagnosis
Barua et al.	2018	X-linked glomerulopathy due to COL4A5 founder variant
Becknell et al.	2011	Novel X-linked glomerulopathy is associated with a COL4A5 missense mutation in a non-collagenous interruption
Chatterjee et al.	2013	Targeted exome sequencing integrated with clinicopathological information reveals novel and rare mutations in atypical, suspected and unknown cases of Alport syndrome or proteinuria
Chen et al.	2021	Functional assessment of a novel COL4A5 splicing site variant in a Chinese X-linked Alport syndrome family
Chen et al.	2016	A novel splicing mutation identified in a Chinese family with X-linked Alport syndrome using targeted next-generation sequencing
Chiereghin et al.	2017	Alport syndrome cold cases: Missing mutations identified by exome sequencing and functional analysis
Choi et al.	2019	Possible digenic disease in a caucasian family with COL4A3 and COL4A5 mutations
Daga et al.	2019	Non-collagen genes role in digenic Alport syndrome
Demosthenous et al.	2012	X-linked Alport syndrome in Hellenic families: Phenotypic heterogeneity and mutations near interruptions of the collagen domain in COL4A5
Ellison	2009	Novel human pathological mutations. Gene symbol: COL4A5. Disease: Alport syndrome
Fallerini et al.	2017	Alport syndrome: Impact of digenic inheritance in patients management
Fernandez-Rosado et al.	2015	Improved genetic counseling in Alport syndrome by new variants of COL4A5 gene
Frese et al.	2019	Kidney injury by variants in the COL4A5 gene aggravated by polymorphisms in slit diaphragm genes causes focal segmental glomerulosclerosis
Fu et al.	2016	X-linked Alport syndrome associated with a synonymous p.Gly292Gly mutation alters the splicing donor site of the type IV collagen alpha chain 5 gene
Fu et al.	2016	Somatic mosaicism and variant frequency detected by next-generation sequencing in X-linked Alport syndrome
Gast et al.	2016	Collagen (COL4A) mutations are the most frequent mutations underlying adult focal segmental glomerulosclerosis
Gibson et al.	2013	Exome analysis resolves differential diagnosis of familial kidney disease and uncovers a potential confounding variant
Gong et al.	2021	Novel mutations of COL4A5 identified in Chinese families with X-linked Alport syndrome and literature review
Gribouval et al.	2018	Identification of genetic causes for sporadic steroid-resistant nephrotic syndrome in adults
Han et al.	2019	De novo mutations in COL4A5 identified by whole exome sequencing in 2 girls with Alport syndrome in Korea

Authors	Year	Title
Helle et al.	2020	Low frequency of parental mosaicism in de novo COL4A5 mutations in X-linked Alport syndrome
Hines et al.	2018	Novel variants in COL4A4 and COL4A5 are rare causes of FSGS in two unrelated families
Hoischen et al.	2009	Array-CGH in unclear syndromic nephropathies identifies a microdeletion in Xq22.3-q23
Höpker et al.	2009	Atypical Alport syndrome associated with a novel COL4A5 mutation
Horinouchi et al.	2020	Pathogenic evaluation of synonymous COL4A5 variants in X-linked Alport syndrome using a minigene assay
Iijima et al.	2010	Severe Alport syndrome in a young woman caused by a t(X;1)(q22.3;p36.32) balanced translocation
Jais et al.	2003	X-linked Alport syndrome: Natural history and genotype-phenotype correlations in girls and women belonging to 195 families: A "European Community Alport Syndrome Concerted Action" study
Kaneko et al.	2010	A family with X-linked benign familial hematuria
Kashiwagi et al.	2019	A family case of X-linked Alport syndrome patients with a novel variant in COL4A5
Komatsu et al.	2019	De novo X-linked Alport syndrome in a 3-year-old girl
Kovács et al.	2016	Efficient targeted next generation sequencing-based workflow for differential diagnosis of Alport-related disorders
Li et al.	2018	Novel deletion mutation in a Chinese family with X-linked Alport syndrome
Li et a.	2019	Identification of a novel COL4A5 mutation in the proband initially diagnosed as IgAN from a Chinese family with X-linked Alport syndrome
Lin et al.	2014	Whole exome sequencing reveals novel COL4A3 and COL4A4 mutations and resolves diagnosis in Chinese families with kidney disease
Lu et al.	2022	Multicenter study on the genetics of glomerular diseases among southeast and south Asians: Deciphering Diversities - Renal Asian Genetics Network (DRAGoN)
Lv et al.	2020	Comparative functional analysis in vitro of 2 COL4A5 splicing mutations at the same site in 2 unrelated Alport syndrome Chinese families
Ma et al.	2011	Twenty-one novel mutations identified in the COL4A5 gene in Chinese patients with X-linked Alport's syndrome confirmed by skin biopsy
Macheroux et al.	2019	The hypomorphic variant p.(Gly624Asp) in COL4A5 as a possible cause for an unexpected severe phenotype in a family with X-linked Alport syndrome
Malone et al.	2017	Functional assessment of a novel COL4A5 splice region variant and immunostaining of plucked hair follicles as an alternative method of diagnosis in X-linked Alport syndrome
Massella et al.	2003	Epidermal basement membrane α5(IV) expression in females with Alport syndrome and severity of renal disease
Mastrangelo et al.	2020	X-linked Alport syndrome in women: Genotype and clinical course in 24 cases
Mencarelli et al.	2015	Evidence of digenic inheritance in Alport syndrome
Mohammad et al.	2014	A female with X-linked Alport syndrome and compound heterozygous COL4A5 mutations
Morais et al.	2022	Kidney organoids recapitulate human basement membrane assembly in health and disease
Morinière et al.	2014	Improving mutation screening in familial hematuric nephropathies through next generation sequencing
Mothes et al.	2002	Alport syndrome associated with diffuse leiomyomatosis: COL4A5-COL4A6 deletion associated with a mild form of Alport nephropathy
Nabais Sá et al.	2013	Deletion of the 5'exons of COL4A6 is not needed for the development of diffuse leiomyomatosis in patients with Alport syndrome

Authors	Year	Title
Neri & Sebastio	2005	Gene symbol: COL4A5. Disease: Alport syndrome
Nozu et al.	2008	Detection of large deletion mutations in the COL4A5 gene of female Alport syndrome patients
Nozu et al.	2017	Characterization of contiguous gene deletions in COL4A6 and COL4A5 in Alport syndrome-diffuse leiomyomatosis
Nozu et al.	2014	X-linked Alport syndrome caused by splicing mutations in COL4A5
Oduware et al.	2021	A case report of COL4A5 gene mutation Alport syndrome in 2 native African children
Oh et al.	2019	Novel mutations in patients with X-linked Alport syndrome: Two case reports
Okamoto et al.	2019	Germline mosaicism is a pitfall in the diagnosis of “sporadic” X-linked Alport syndrome
Palenzuela et al.	2002	A new point mutation in the COL4A5 gene described in a Spanish family with X-linked Alport syndrome
Papazachariou et al.	2017	Frequent COL4 mutations in familial microhematuria accompanied by later-onset Alport nephropathy due to focal segmental glomerulosclerosis
Shi et al.	2021	Case report: Preimplantation genetic testing and pregnancy outcomes in women with Alport syndrome
Šlajpah et al.	2007	Sixteen novel mutations identified in COL4A3, COL4A4, and COL4A5 genes in Slovenian families with Alport syndrome and benign familial hematuria
Strasser et al.	2012	COL4A5-associated X-linked Alport syndrome in a female patient with early inner ear deafness due to a mutation in MYH9
Sun et al.	2021	Determination of the pathogenicity of a novel COL4A5 missense variant by CRISPR-Cas9 in kidney podocytes
Tang et al.	2019	An overlap of Alport syndrome and rheumatoid arthritis in a patient and literature review
Tug et al.	2011	Linkage analysis and a novel COL4A5 mutation in a large Turkish family with Alport syndrome
Uliana et al.	2011	Alport syndrome and leiomyomatosis: The first deletion extending beyond COL4A6 intron 2
Voskarides et al.	2018	COL4A5 and LAMA5 variants co-inherited in familial hematuria: Digenic inheritance or genetic modifier effect?
Wang et al.	2002	Phenotypic and genotypic features of Alport syndrome in Chinese children
Wang et al.	2020	The first COL4A5 exon 41A glycine substitution in a family with Alport syndrome
Wang et al.	2021	Combination of a novel genetic variant in CFB gene and a pathogenic variant in COL4A5 gene in a sibling renal disease: A case report
Wang et al.	2007	Correlation between mRNA expression level of the mutant COL4A5 gene and phenotypes of XLAS females
Wang et al.	2004	Effect of glycine substitutions on α5(IV) chain structure and structure-phenotype correlations in Alport syndrome
Weber et al.	2016	Identification of 47 novel mutations in patients with Alport syndrome and thin basement membrane nephropathy
Wilson et al.	2007	A novel Cys1638Tyr NC1 domain substitution in α5(IV) collagen causes Alport syndrome with late onset renal failure without hearing loss or eye abnormalities
Wu et al.	2021	A disease-causing variant of COL4A5 in a Chinese family with Alport syndrome: A case series
Wuttke et al.	2015	A COL4A5 mutation with glomerular disease and signs of chronic thrombotic microangiopathy
Xiu et al.	2014	A novel COL4A5 mutation identified in a Chinese Han family using exome sequencing
Xu et al.	2022	New COL4A5 mutation in IgA nephropathy

Authors	Year	Title
Yamamura et al.	2017	Natural history and genotype–phenotype correlation in female X-Linked Alport syndrome
Zhang et al.	2020	An overview of the multi-pronged approach in the diagnosis of Alport syndrome for 22 children in Northeast China
Zhao et al.	2012	A novel splice site mutation in the COL4A5 gene in a Chinese female patient with rare ocular abnormalities
Zhao et al.	2019	Novel mutations of COL4A3, COL4A4, and COL4A5 genes in Chinese patients with Alport syndrome using next generation sequence technique
Zhao et al.	2020	Identification of four novel mutations in the COL4A5 gene identified in Chinese patients with X-linked Alport syndrome
Zhou et al.	2021	Clinical manifestations of Alport syndrome-diffuse leiomyomatosis patients with contiguous gene deletions in COL4A6 and COL4A5
Zhu et al.	2020	A novel frameshift mutation of COL4A5 in a Chinese family with presumed IgA nephropathy and chronic glomerulonephritis
Zupan et al.	2020	Mutations in collagen genes in the context of an isolated population

Supplementary Table S4. Geographic location of studies included in this systematic review

Location[a]	Articles included (% of total)
**Africa**	**1 (1%)**
Nigeria	1 (1%)
**Americas**	**7 (8%)**
Canada	1 (1%)
United States	6 (7%)
**Asia**	**44 (49%)**
China	25 (28%)
Japan	13 (15%)
Kazakhstan	1 (1%)
Saudi Arabia	1 (1%)
Singapore	1 (1%)
South Korea	2 (2%)
Turkey	1 (1%)
**Europe**	**35 (39%)**
Cyprus	3 (3%)
Denmark	1 (1%)
France	3 (3%)
Germany	9 (10%)
Hungary	1 (1%)
Italy	8 (9%)
Poland	1 (1%)
Portugal	1 (1%)
Slovenia	2 (2%)
Spain	3 (3%)
United Kingdom	3 (3%)
**Oceania**	**2 (2%)**
Australia	1 (1%)
New Zealand	1 (1%)
**Total**	**89**

[a] Based on institutional affiliation of first author.

Supplementary Table S5. Clinical features and variant types in 274 age-matched females with X-linked Alport syndrome.

	**Proteinuria (n=160)**	**No proteinuria (n=114)**	***p*-value**
**Clinical features**			
Median age (IQR) (years)	25 (14-43) (n=160)	24 (14-42) (n=114)	0.79
Kidney failure (n, %)	37/151 (25%)	1/107 (1%)	**<0.0001**
Hearing loss (n, %)	21/112 (19%)	9/84 (11%)	0.16
Ocular changes (n, %)	12/88 (14%)	2/77 (3%)	**0.01**
**Variant types**			
Truncating and large variants (n, %)	46/160 (29%)	19/114 (17%)	**0.02**
Splicing variants (n, %)	32/160 (20%)	14/114 (12%)	0.10
Missense variants (n, %)	82/160 (51%)	81/114 (71%)	**0.001**

Significant values are in bold; IQR, interquartile range

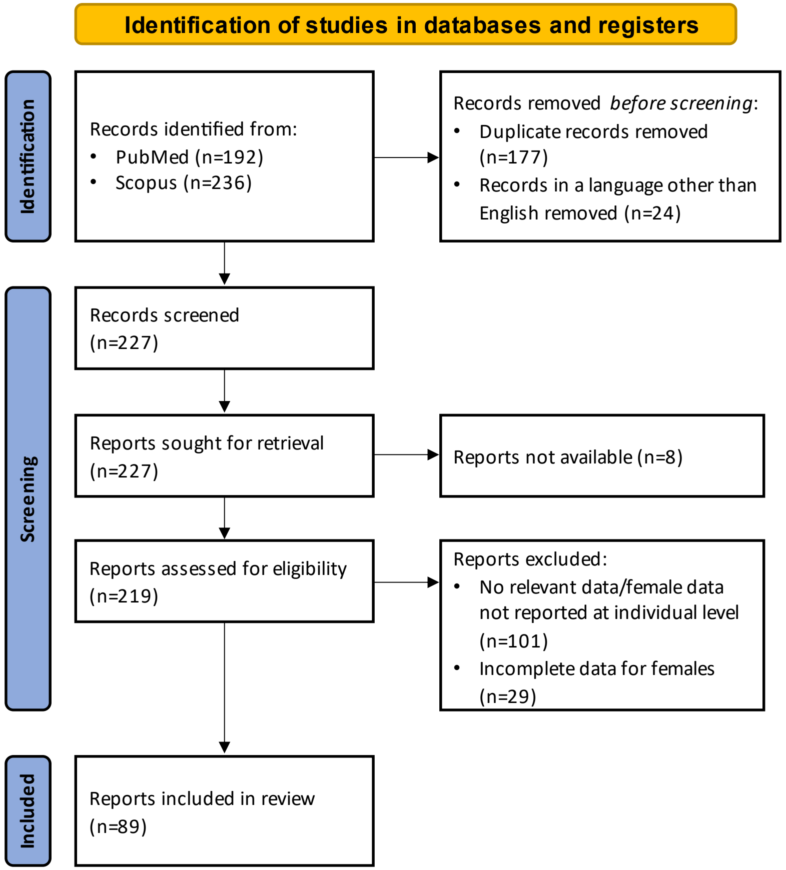

Supplementary Figure S1: PRISMA flow chart for study selection

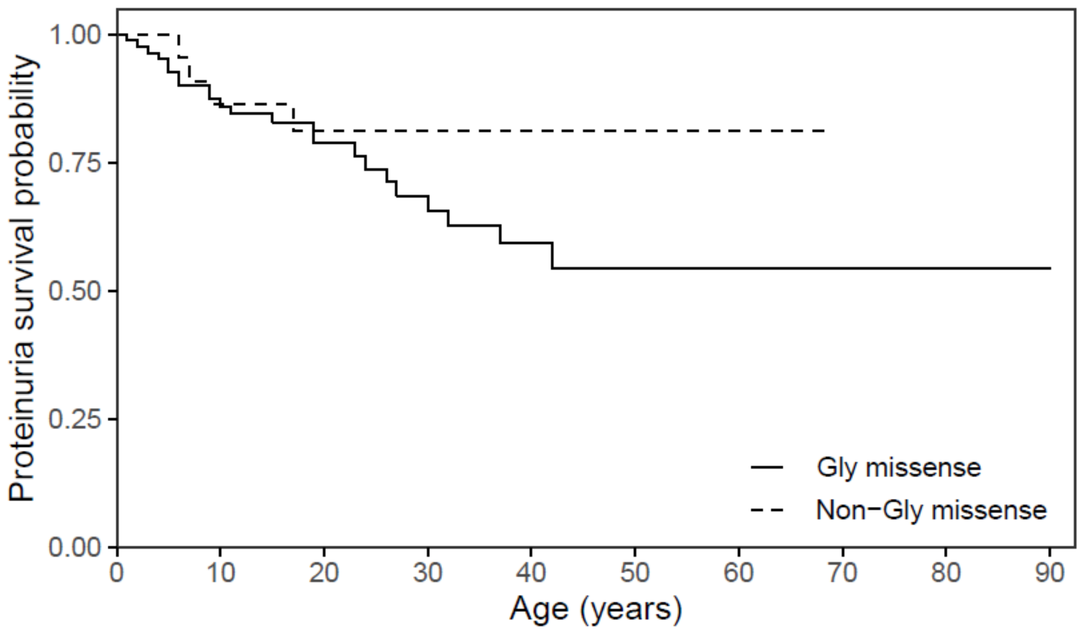

Supplementary Figure S2. Proportion of females without proteinuria, stratified by missense variant type (p=0.12). Gly missense variants (n_{total}=83, n_{prot}=23); non-Gly missense variants (n_{total}=25, n_{prot}=4). Neither survival curve passes below 0.5, so the median ages of survival are not available. Censored data points are not shown.

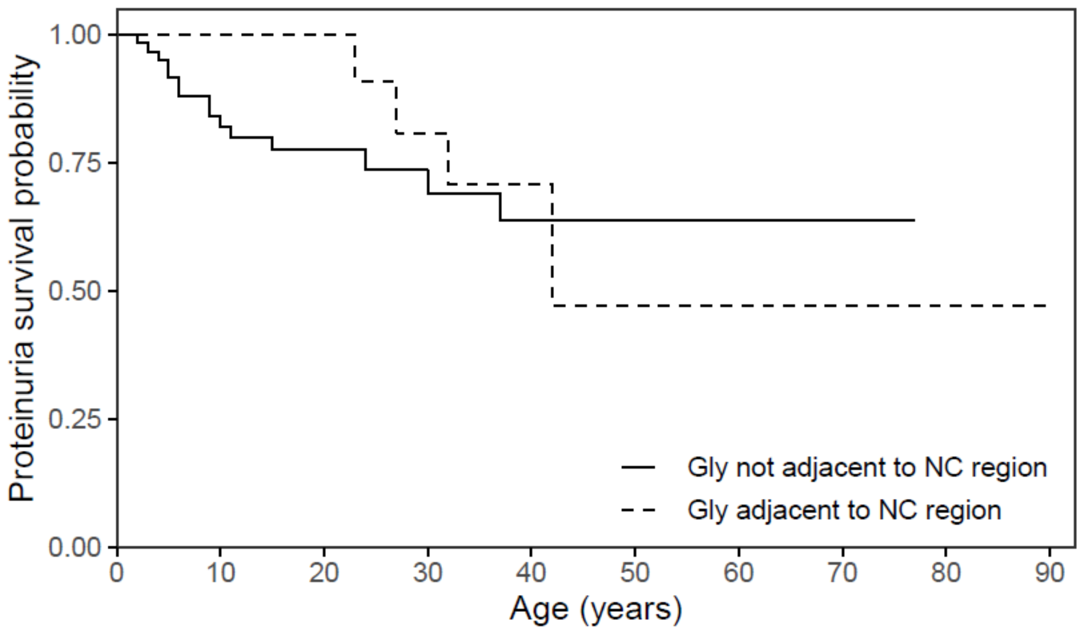

Supplementary Figure S3. Proportion of females without proteinuria, stratified by location of Gly substitution ($p=0.45$). Gly not adjacent to NC region ($n_{total}=60$, $n_{prot}=15$); Gly adjacent to NC region ($n_{total}=19$, $n_{prot}=4$, median=42 years). The 'Gly not adjacent to NC region' survival curve does not pass below 0.5, so the median age of survival is not available. Censored data points are not shown. NC, non-collagenous.

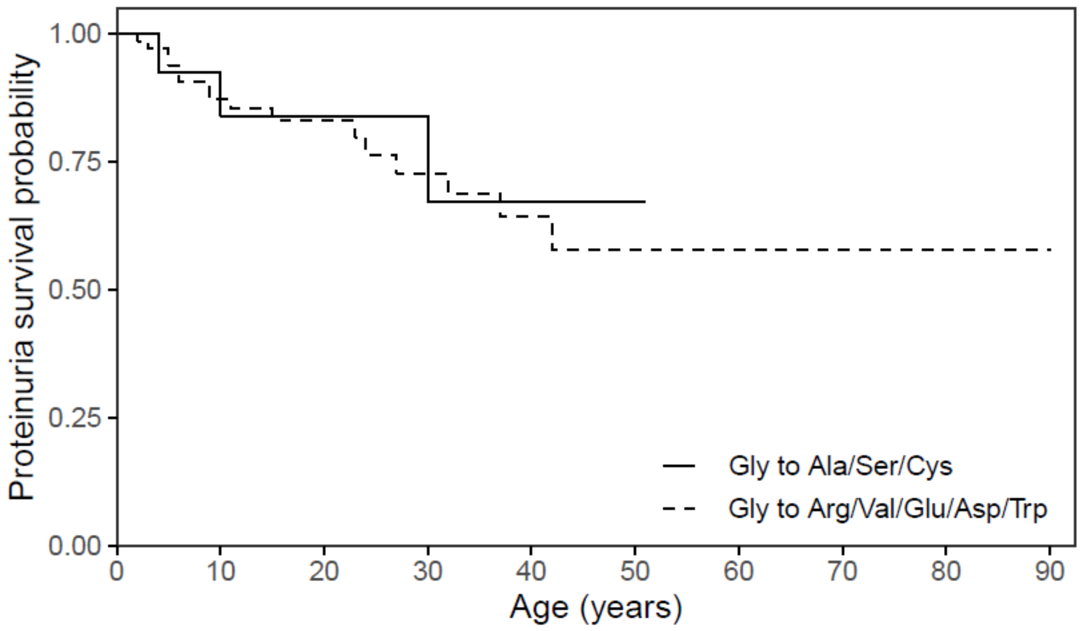

Supplementary Figure S4. Proportion of females without proteinuria, stratified by the relative instability of the residue replacing Gly (p=0.83). Gly to Ala, Ser or Cys (n_{total}=13, n_{prot}=3); Gly to Arg, Val, Glu, Asp or Trp (n_{total}=66, n_{prot}=16). Neither survival curve passes below 0.5, so the median ages of survival are not available. Ala, Ser and Cys were considered to be mildly destabilising residues. Arg, Val, Glu, Asp and Trp were considered to be highly destabilising residues. Censored data points are not shown.

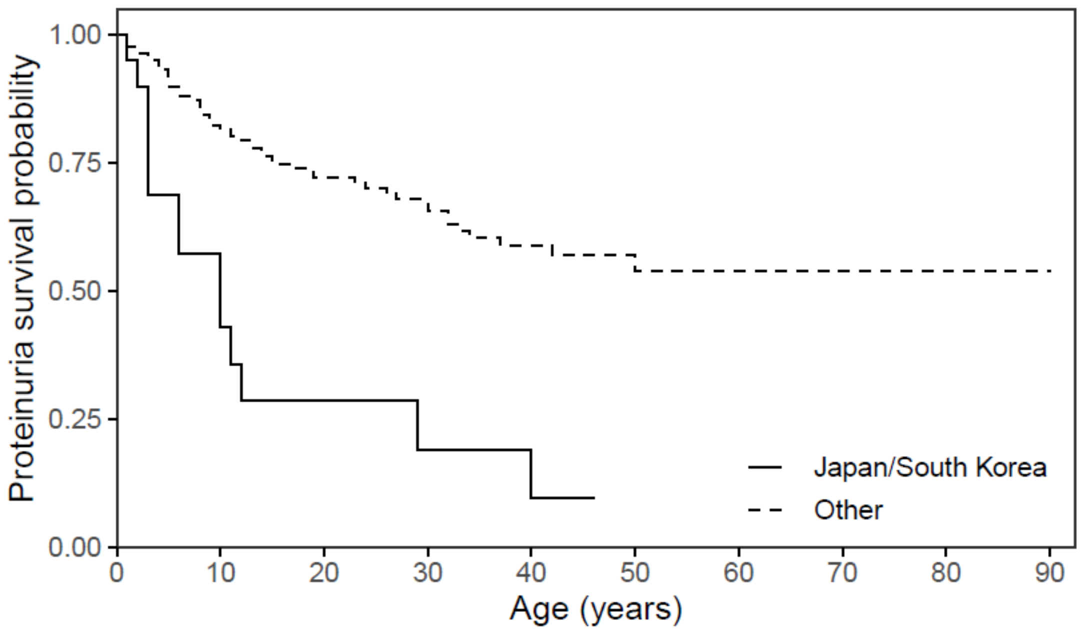

Supplementary Figure S5. Proportion of females without proteinuria, stratified by location of study ($p<0.0001$). Japan/South Korea ($n_{total}=20$, $n_{prot}=14$, median=10 years); other ($n_{total}=161$, $n_{prot}=53$). The 'other' survival curve does not pass below 0.5, so the median age of survival is not available. Japan and South Korea both have mass urinary screening programmes for school-aged children. Censored data points are not shown.

PRISMA checklist

Section and Topic	Item #	Checklist item	Location where item is reported
**TITLE**			
Title	1	Identify the report as a systematic review.	Page 1
**ABSTRACT**			
Abstract	2	See the PRISMA 2020 for Abstracts checklist.	Page 2
**INTRODUCTION**			
Rationale	3	Describe the rationale for the review in the context of existing knowledge.	Page 4
Objectives	4	Provide an explicit statement of the objective(s) or question(s) the review addresses.	Page 4
**METHODS**			
Eligibility criteria	5	Specify the inclusion and exclusion criteria for the review and how studies were grouped for the syntheses.	Page 5
Information sources	6	Specify all databases, registers, websites, organisations, reference lists and other sources searched or consulted to identify studies. Specify the date when each source was last searched or consulted.	Page 5
Search strategy	7	Present the full search strategies for all databases, registers and websites, including any filters and limits used.	Supp T1-2
Selection process	8	Specify the methods used to decide whether a study met the inclusion criteria of the review, including how many reviewers screened each record and each report retrieved, whether they worked independently, and if applicable, details of automation tools used in the process.	Page 5
Data collection process	9	Specify the methods used to collect data from reports, including how many reviewers collected data from each report, whether they worked independently, any processes for obtaining or confirming data from study investigators, and if applicable, details of automation tools used in the process.	Page 5
Data items	10a	List and define all outcomes for which data were sought. Specify whether all results that were compatible with each outcome domain in each study were sought (e.g. for all measures, time points, analyses), and if not, the methods used to decide which results to collect.	Page 5
	10b	List and define all other variables for which data were sought (e.g. participant and intervention characteristics, funding sources). Describe any assumptions made about any missing or unclear information.	Page 5
Study risk of bias assessment	11	Specify the methods used to assess risk of bias in the included studies, including details of the tool(s) used, how many reviewers assessed each study and whether they worked independently, and if applicable, details of automation tools used in the process.	N/A
Effect measures	12	Specify for each outcome the effect measure(s) (e.g. risk ratio, mean difference) used in the synthesis or presentation of results.	Page 6
Synthesis methods	13a	Describe the processes used to decide which studies were eligible for each synthesis (e.g. tabulating the study intervention characteristics and comparing against the planned groups for each synthesis (item #5)).	Page 6
	13b	Describe any methods required to prepare the data for presentation or synthesis, such as handling of missing summary statistics, or data conversions.	Page 5
	13c	Describe any methods used to tabulate or visually display results of individual studies and syntheses.	Page 6
	13d	Describe any methods used to synthesize results and provide a rationale for the choice(s). If meta-analysis was performed, describe the model(s), method(s) to identify the presence and extent of statistical heterogeneity, and software package(s) used.	Page 6
	13e	Describe any methods used to explore possible causes of heterogeneity among study results (e.g. subgroup analysis, meta-regression).	Pages 7-8
	13f	Describe any sensitivity analyses conducted to assess robustness of the synthesized results.	N/A
Reporting bias assessment	14	Describe any methods used to assess risk of bias due to missing results in a synthesis (arising from reporting biases).	N/A
Certainty assessment	15	Describe any methods used to assess certainty (or confidence) in the body of evidence for an outcome.	N/A

Section and Topic	Item #	Checklist item	Location where item is reported
**RESULTS**			
Study selection	16a	Describe the results of the search and selection process, from the number of records identified in the search to the number of studies included in the review, ideally using a flow diagram.	Fig 1
	16b	Cite studies that might appear to meet the inclusion criteria, but which were excluded, and explain why they were excluded.	N/A
Study characteristics	17	Cite each included study and present its characteristics.	Supp T3
Risk of bias in studies	18	Present assessments of risk of bias for each included study.	N/A
Results of individual studies	19	For all outcomes, present, for each study: (a) summary statistics for each group (where appropriate) and (b) an effect estimate and its precision (e.g. confidence/credible interval), ideally using structured tables or plots.	N/A
Results of syntheses	20a	For each synthesis, briefly summarise the characteristics and risk of bias among contributing studies.	Pages 7-8
	20b	Present results of all statistical syntheses conducted. If meta-analysis was done, present for each the summary estimate and its precision (e.g. confidence/credible interval) and measures of statistical heterogeneity. If comparing groups, describe the direction of the effect.	Pages 7-8
	20c	Present results of all investigations of possible causes of heterogeneity among study results.	Pages 7-8
	20d	Present results of all sensitivity analyses conducted to assess the robustness of the synthesized results.	N/A
Reporting biases	21	Present assessments of risk of bias due to missing results (arising from reporting biases) for each synthesis assessed.	N/A
Certainty of evidence	22	Present assessments of certainty (or confidence) in the body of evidence for each outcome assessed.	N/A
**DISCUSSION**			
Discussion	23a	Provide a general interpretation of the results in the context of other evidence.	Pages 9-10
	23b	Discuss any limitations of the evidence included in the review.	Pages 9-10
	23c	Discuss any limitations of the review processes used.	Pages 9-10
	23d	Discuss implications of the results for practice, policy, and future research.	Page 10
**OTHER INFORMATION**			
Registration and protocol	24a	Provide registration information for the review, including register name and registration number, or state that the review was not registered.	Page 11
	24b	Indicate where the review protocol can be accessed, or state that a protocol was not prepared.	Page 11
	24c	Describe and explain any amendments to information provided at registration or in the protocol.	N/A
Support	25	Describe sources of financial or non-financial support for the review, and the role of the funders or sponsors in the review.	Page 11
Competing interests	26	Declare any competing interests of review authors.	Page 11
Availability of data, code and other materials	27	Report which of the following are publicly available and where they can be found: template data collection forms; data extracted from included studies; data used for all analyses; analytic code; any other materials used in the review.	Page 11

From: Page MJ, McKenzie JE, Bossuyt PM, Boutron I, Hoffmann TC, Mulrow CD, et al. The PRISMA 2020 statement: an updated guideline for reporting systematic reviews. BMJ 2021;372:n71. doi: 10.1136/bmj.n71

For more information, visit: http://www.prisma-statement.org/
